# Fear expressions of dogs during New Year fireworks: a video analysis

**DOI:** 10.1038/s41598-020-72841-7

**Published:** 2020-09-29

**Authors:** Sarah Gähwiler, Annika Bremhorst, Katinka Tóth, Stefanie Riemer

**Affiliations:** 1 Companion Animal Behaviour Group, Division of Animal Welfare, Vetsuisse Faculty, University of Bern, Bern, Switzerland; 2 Animal Behaviour Cognition and Welfare Research Group, School of Life Sciences, University of Lincoln, Lincoln, UK; 3 MTA Research Centre for Natural Sciences, Institute of Cognitive Neuroscience and Psychology, Budapest, Hungary

**Keywords:** Zoology, Animal behaviour

## Abstract

A high proportion of pet dogs show fear-related behavioural problems, with noise fears being most prevalent. Nonetheless, few studies have objectively evaluated fear expression in this species. Using owner-provided video recordings, we coded behavioural expressions of pet dogs during a real-life firework situation at New Year’s Eve and compared them to behaviour of the same dogs on a different evening without fireworks (control condition), using Wilcoxon signed ranks tests. A backwards-directed ear position, measured at the base of the ear, was most strongly associated with the fireworks condition (effect size: Cohen’s d = 0.69). Durations of locomotion (d = 0.54) and panting (d = 0.45) were also higher during fireworks than during the control condition. Vocalisations (d = 0.40), blinking (d = 0.37), and hiding (d = 0.37) were increased during fireworks, but this was not significant after sequential Bonferroni correction. This could possibly be attributed to the high inter-individual variability in the frequency of blinking and the majority of subjects not vocalising or hiding at all. Thus, individual differences must be taken into account when aiming to assess an individual’s level of fear, as relevant measures may not be the same for all individuals. Firework exposure was not associated with an elevated rate of other so-called ‘stress signals’, lip licking and yawning.

## Introduction

Emotions are short-term affective states that are elicited by internal or external events and comprise changes in several components, including neuro-physiological, cognitive (appraisal), motivational and expression components, as well as the subjective experience, or feeling^1,2^. As nonhuman animals cannot report on what they are feeling, we can attempt to infer the emotion that is likely being experienced based on changes in these emotion components (e.g. physiological stress responses, behavioural expression and behavioural choices) and the circumstances in which these are occurring^1,3^. For instance, behaviour and expressions during exposure to potential threats can be assumed to be fear-related while those in response to denial of access to a desirable stimulus can be interpreted as frustration (c.f.^4,5^).

Identifying indicators of emotions in nonhuman animals is important for assessing their welfare state as well as to improve our ability to predict subsequent behavioural responses. This is particularly relevant in domestic dogs, which live more closely with us than any other species and do not only play a role as social partners^6,7^, but also as therapy dogs^8^, assistance dogs^9,10^ or working dogs with functions ranging from search and rescue^11^ to police work^12^. Nonetheless, problems due to fear-related behaviour are often reported in both pet and working dogs (e.g.^13^; reviewed in^14^), and owners may even underestimate fear in their dogs when not questioned about the specific signs exhibited^15^.

Fear is a key emotion that is highly adaptive by prompting animals to react adequately to threat^16^. When an environmental threat (i.e. a stressor) is perceived, the brain’s fear system is activated, initiating behavioural and physiological stress responses^17^. Neuroscientists have identified distinctions between ‘fear’ (an adaptive response to a stimulus considered to be potentially dangerous) and ‘anxiety’ (anticipation of a negative outcome, lacking a specific eliciting stimulus)^18,19^. However, in practice it is often not easy to distinguish between the two concepts behaviourally, especially considering that anxiety promotes fear and vice versa^18,20^. Hereafter, we shall use the term ‘fear’ to denote both fear and anxiety responses in dogs, since they have in common that they are aversive emotional states due to a (perceived) threat that are associated with intense negative emotions and physiological changes^21^, and sufficient criteria to distinguish between the different concepts in dogs have yet to be established (c.f.^22^).

In response to fear, animals may respond by freezing, hiding or fleeing, depending on the distance of the threat and the perceived likelihood of success^23^. Congruent with the internal state, animals may also show specific expressions; for example, a backward position of the ears has been associated with fear in a variety of mammalian species including sheep^24^, goats^25^, pigs^26^, horses^27,28^ and cats^29^. Despite the fear response being evolutionarily adaptive, fear may often be elicited in situations that do not pose real threats, as a ‘false negative’ may entail high fitness costs (risk of death) compared to a ‘false positive’ (an over-reaction to an actually harmless stimulus)^30^. Being associated with a negative affective state, fear responses to stimuli in a human-dominated environment may have detrimental effects on animals’ welfare, without serving a function in aiding survival. For example, a fear of loud noises is innate in many animals^20^, and noise fears are among the most common fears affecting pet dogs^15,31,32^. It is important to recognise these negative affective states, to address them and to use the right measures to evaluate treatment progress.

Surprisingly, there is a relative lack of studies objectively evaluating the expression of emotions in dogs. A few controlled studies have measured behaviour and expression in dogs when exposed to putatively fear-inducing stimuli (note that the terminology used was variable, with some authors referring to discrete emotions such as fear^33,34^, others to stress more generally^35–37^). Classic studies by Beerda et al.^36,37^ in which dogs were exposed to various potentially threatening stimuli, indicated that a lowered posture (including the ears, tail and body position) constitutes the most consistent indicator of stress (no reference to emotions was made) of both high and moderate intensity in dogs. Furthermore, Beerda et al.^36^ suggested that oral behaviours (such as frequency of tongue protrusions and snout licking), yawning, body shaking and crouching are indicative of acute stress in dogs. However, as the severity (as judged by the authors) of stressors was confounded with the type of stressor (social/non-social)^36^, no clear conclusions could be drawn whether different observed behaviours were associated with the severity of the stressor or with the social vs non-social context.

More recently, Stellato^34^ confirmed that fear in dogs towards both a social stimulus (sudden appearance of a masked stranger) and a non-social stimulus (sudden appearance of a garbage bag) appears to be associated with avoidance and/or a reduced body posture, whereas effects on more subtle behaviours (such as body shaking, barking, whining, lip licking, yawning, tail wagging and paw lifting) were more inconsistent^34^. Another controlled study on puppies aged under six months inferred potential fear indicators based on their association with avoidance of different non-social stimuli considered to induce mild to moderate fear^33^. On this basis, a lowered posture, a lowered tail, freezing, retreating, flinching, paw lifting and barking were identified as the most reliable fear indicators, while there was no association of avoidance with ear position, tail-wagging and lip licking^33^.

Although noise fears represent the most common fears in dogs^15,32,38^, only few studies have been aimed to objectively quantify fear-related behaviours in dogs during noise events, and all of these used audio recordings. Dreschel and Granger^39^ described excessive salivation, vocalisations, hiding, pacing, panting, remaining near the owner, and trembling in dogs exposed to a thunderstorm recording at the clinic, though they did not assess to which extent these behaviours differed from a baseline condition.

De Souza et al.^40^ found that dogs both classified as ‘sound sensitive’ and as ‘not sound sensitive’ (categorisations based on owners’ description of the severity of signs) reacted to recordings of a firework by increasing alertness and attention, panting, searching for the sound, startling, trembling, hiding and running away, with ‘sound sensitive’ dogs showing a greater intensity of reaction for alertness and attention, searching the sound, trembling, hiding and running away, as well as showing less resting and winking/sleeping. No significant effects were reported for variables related to the posture and tail, lip licking, yawning, vocalisations, elimination, and destruction^40^.

Some experimental studies yielded conflicting behavioural results, reporting either a reduction in activity^41,42^ or an increase in activity^43–45^ in response to audio recordings of thunderstorms or fireworks. These different findings could potentially be explained by different coping strategies of dogs when exposed to fear-inducing noises, an active and a passive response: Landsberg et al.^45^ used separate scores for active (including aimless, repetitive or stereotypic pacing, running or circling; retreating to a hide, digging, climbing, jumping or barking) and passive (encompassing decreased activity, freezing against a wall, staying close to the door, crouching, tail between legs, ears back, panting, trembling, being alert/tense/vigilant, salivating, yawning, lip licking, lifting a foreleg and whining) responses to audio stimulation. Both active and passive scores were increased in beagle dogs during noise exposure compared to before and after stimulation, indicating validity of the scores. However, individual behavioural parameters were not analysed^45^.

While the standardisation of noise exposure in studies using laboratory settings has many advantages, it is not clear to what extent the findings can be transferred to dogs’ fear responses in a home setting. For example, with regard to noise fears, some discrepancies between owner-reported improvements in fearfulness and dogs’ behaviour when exposed to a noise recording in a clinic setting have been reported^46,47^. Recordings cannot fully represent the characteristics of real noises and the range of stimuli (e.g. atmospheric changes associated with thunder or lights accompanying fireworks) (c.f.^48,49^), and some dogs do not respond to these simulations even under optimal conditions^49^.

Here we aimed to identify fear indicators in pet dogs in a real-life situation, during New Year’s eve fireworks, by comparing dogs’ behavioural expression during firework exposure to a control night with no fireworks present. By taking advantage of situations in which dogs are exposed unavoidably to these stimuli, i.e. at New Year’s Eve, it was possible to study dogs’ fear reactions without ethical concerns due to an artificial induction of fears. Hereafter we refer to dogs’ expressions during fireworks as ‘fear’ expressions, being aware that we can only infer the underlying affective state.

### Predictions

Based on the literature, we predicted that dogs show the following behavioural expressions during fireworks compared to the control condition: a lowered posture (c.f.^33–36^), a lowered tail position (c.f.^33,45^), a more backwards-directed ear position (c.f.^45,50^), an increased frequency of vocalisations (c.f.^15,39^), an increased duration of panting (c.f.^22,39,51^), an increased frequency of blinking (c.f.^52^), an increased frequency of lip licking and yawning (c.f.^36,37^), a change in activity (time moving, c.f.^43,45^) and an increased time hiding (c.f.^40^).

To test these predictions, we performed a citizen science study, asking volunteer dog owners to film their dogs for five minutes (1) during fireworks on New Year’s Eve (firework video) and (2) again several days later at a similar time in the evening (control video), when they considered their dog’s behaviour to have normalised. Videos of 36 dogs were coded based on an ethogram (Table 1), and expressions and behaviours during firework and control videos were compared using Wilcoxon signed ranks tests. In an accompanying questionnaire, owners rated on a scale from 1 to 5 how much their dogs’ welfare was impaired by fireworks (‘Welfare Impaired score’, c.f.^32^). Only dogs with scores of 2 and above were considered in the current study (Supplementary Table S1). Although we excluded dogs receiving medication, and thus potentially the most fearful subjects, the median ‘Welfare Impaired score’ of the 36 dogs included was still high at 4 (IQR 2.75–5).

**Table 1:** Definition of coded behaviours.

Variable grouping	Variable name	Definition	Final variable in analysis
**Frequencies**			
Events	Blink	Eyes closed at least 80% for not more than one second	Blink
	Snout lick	Tongue licks over lips or nose; not counted during 5 s after eating	Lip lick (sum of snout lick and lip smack)
	Lip smack	Mouth slightly opened with a slight protrusion of the tongue, not counted during 5 s after eating	
	Yawn	Mouth wide open for at least one second	Yawn
	Whine/howl	Sustained sound through semi closed jaws	Vocalisation (sum of whine/howl and bark)
	Bark	Abrupt, harsh sound associated with an opened mouth	
**Point sampling**			
Gross behaviours	Move	A movement by the limbs leading to a change in the location of the dog’s body	Proportion of time points moving
	Hide	At least two thirds of the dog’s body are beneath or behind something	Proportion of time points hiding
**Durations**			
Durations	Pant	Rapid respiration through opened mouth	Pant
	Sleep	Lying with the eyes closed	
	Invisible	Whole dog not in view or too dark to evaluate posture and behaviour	
	Body invisible	Evaluation of body posture not possible, but head visible	
	Head invisible	Mouth and eyes not visible; ears can be visible. (This variable was only used to calculate the correct frequency of oral behaviours and blinking per minute visible. Ear position was scored separately via instantaneous sampling and a NA score was given if the ears were not visible)	
	Eyes invisible	Both eyes not visible or video too dark to observe any blinking	
	Mouth invisible	Mouth not visible or filmed from just above so that oral behaviour such as yawning are not observable	
**Instantaneous sampling**			
Posture scores	Ear position	Measured at the base of the ear, not coded in sleeping dogs. 1 = turned as far backwards as possible, 3 = neutral, 5 = directed forward. 2 and 4: intermediate scores. If one ear is turned further back than the other, the scale is used for the ear that is further back	Ear position
	Tail position	Score from 1 to 5, can only be assessed whilst dog is standing 1 = tail tucked in underneath the belly, 3 = neutral, 5 = highest possible tail carriage depending on breed/type. 2 and 4: intermediate scores	Tail position
	Body posture	Score from 1 to 5, can only be assessed whilst dog is standing. 1 = cowering, 3 = neutral, 5 = leaning forward. 2 and 4: intermediate scores	Body posture

## Results

**Figure 1:**
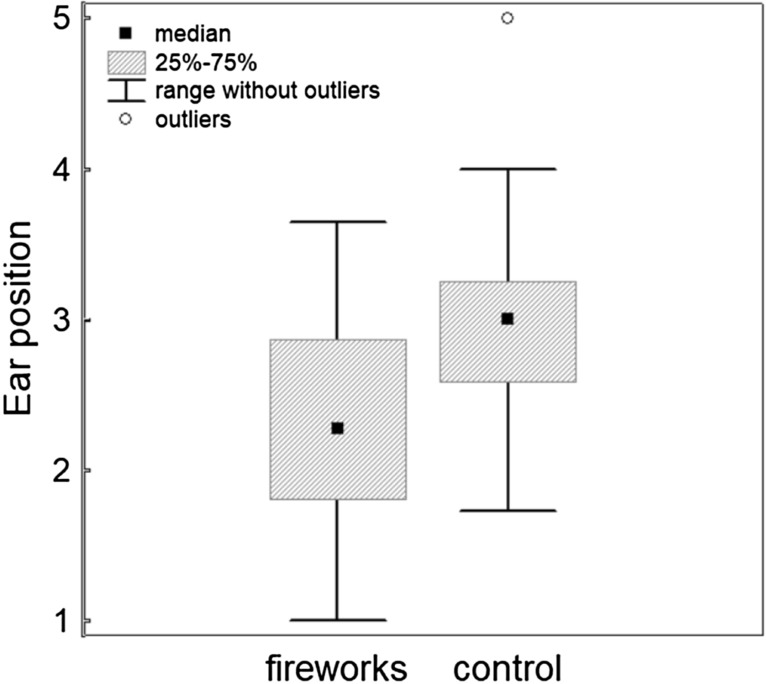
Median, interquartile range, 5–95% range and outliers of dogs’ ear position during the firework and control condition, respectively, with a score of 1 denoting the most backward ear position and a score of 5 the most forward ear position.

**Figure 2:**
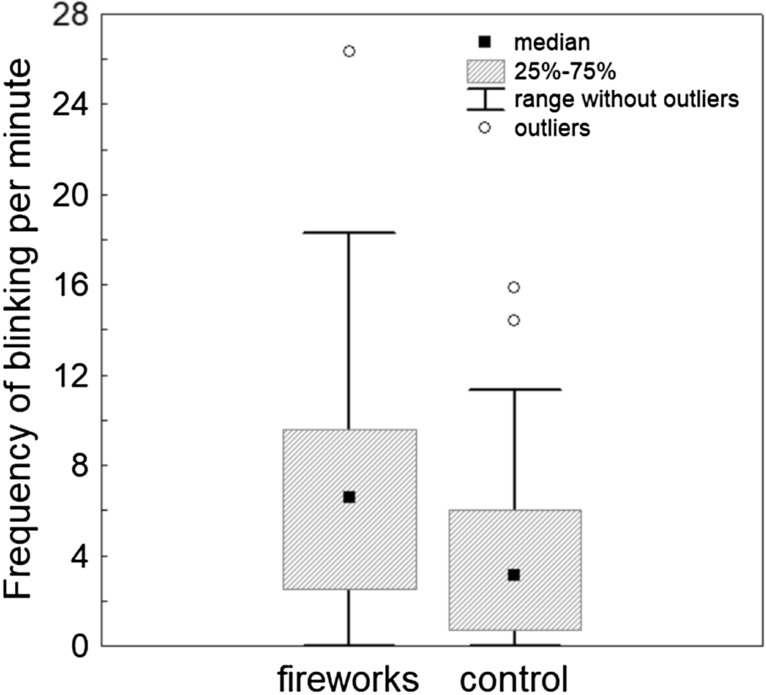
Median, interquartile range, 5–95% range and outliers of frequency of blinking per minute during the firework and control condition, respectively.

**Figure 3:**
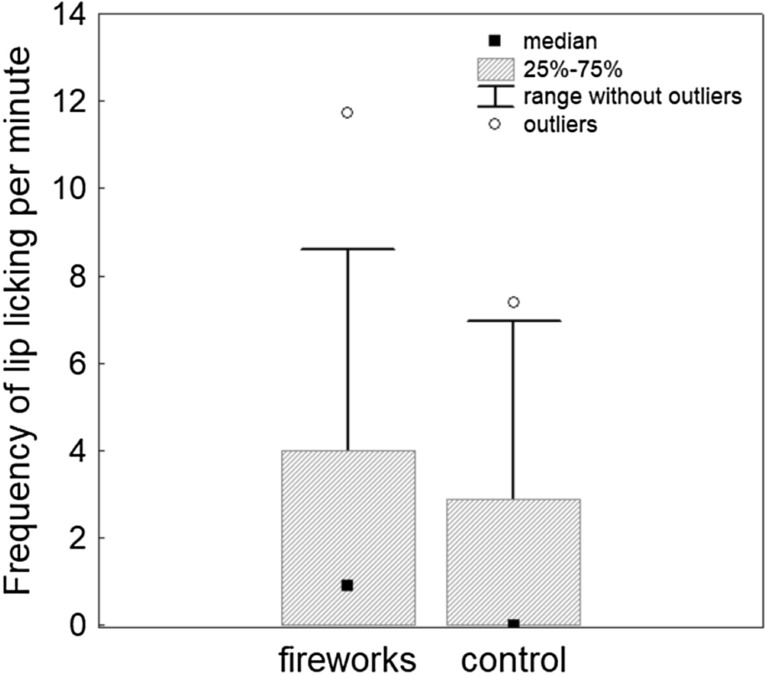
Median, interquartile range, range and outliers of frequency of lip licking per minute during the firework and control condition, respectively.

The behavioural comparison between the fireworks condition and the control condition demonstrated a highly significant difference in ear position (scored at the base of the ears from 1 to 5), with fireworks being associated with more backwards-directed ears (Wilcoxon signed ranks test, Z = 4.05, p = 0.0005, Cohen’s d = 0.68, Fig. 1, Supplementary Table S2). Dogs were furthermore moving (Z = 3.24, p = 0.001, d = 0.54) and panting (Z = 2.67, p = 0.008, d = 0.45) significantly more during fireworks. Vocalisations (Z = 2–37, p = 0.018, d = 0.40), blinking (Z = 2.23, p = 0.026, d = 0.37, Fig. 2) and hiding (Z = 2.2, p = 0.028, d = 0.37) were also increased during fireworks compared to the control videos, but this was no longer significant when controlling for multiple testing (Supplementary Table S2). Neither the frequency of lip licking (Z = 0.74, p = 0.46, d = 0.13, Fig. 3) nor the frequency of yawning (Z = 1.22, p = 0.221, d = 0.21) differed significantly between the two conditions.

The variables ‘body posture’ and ‘tail position’ could not be included in the analysis, as dogs were mostly in a lying position during the control condition, so data from both conditions were available for only five (‘body posture) and three (‘tail position’) out of the 36 subjects, respectively.

## Discussion

In the current study, a backwards directed ear position was the indicator most strongly associated with the firework condition, and presumably a fearful state in dogs, perhaps because tail position and body posture could not be evaluated. Although “ears back” or “flattened ears” are often used to assess fearfulness in dogs (e.g.^43,45,48^), the scientific data to validate this expression as an indicator of fear is surprisingly thin. In studies by Beerda et al.^36,37^, a ‘posture score’ comprising ear position along with tail position and breed specific posture was one of the best indicators of both “moderate and severe stress”^36,37^; however, even if the individual components of this score, body position, tail position and ear position often co-vary, it is important to have more detailed measures available and to take into account evidence from individual variables.

Flint et al.^33^ differentiated between a lowered posture, a lowered tail and “ears back” in their study on fear expression in puppies, but while their study confirmed both a reduced posture and a lowered tail to be indicative of fear in dogs, they found no relationship between puppies’ avoidance behaviour (used as proxy to infer fearfulness) and whether they had their ears back. This could simply be explained by the low video quality, as suggested by Flint et al.^33^, but another possible explanation could be the confounding effects of alertness. In the firework videos in the current study, dogs often appeared to be vigilant and may thus have been holding the ears up. However, when looking at the base of the ears (as per our definition), it was discernible that the ears were turned backwards relative to the position judged to be neutral by the raters.

Our data thus suggest that a backwards-directed ear position, judged at the base of the ears, is a valid indicator of a fearful state in dogs. In the Dog Facial Action Coding System (DogFACS^53^), a system for objectively analysing facial movements in dogs, a differentiation is made between several ear position changes including the ears flattener, the ears rotator and ears downwards. While the ears flattener and the ears rotator can only be distinguished in dog with upright ears but not those with floppy ears^43^, it is a possibility that it is the backwards rotation of the ears that is most consistently associated with non-social fear at least in dogs with erect ears. Nonetheless, while fear of fireworks appears to be reliably associated with backwards-directed ears (as measured at the base of the ears), this is only one of several contexts where this expression may be observed; for example, backwards-directed ears are also shown during frustration^5^, as well as in affiliative greeting situations during active submission^54^. Future studies could investigate subtle differences in ear position and associated contexts in more detail.

Locomotion was significantly elevated during fireworks compared to the control night. While this finding per se does not necessarily signify fear, as dogs might have adapted their own activity to the heightened activity in the household at a time of day where they were usually sleeping, pacing has previously been described as a behavioural expression of noise fears in dogs^22,39^. Our definition did not allow to differentiate between pacing and locomotion, but personal observations indicated that besides pacing, an increase in locomotion appeared to be due to an inability to settle (attempting to lie down in one locations, then changing to a new location shortly thereafter) in some dogs in the current study. Thus it might reflect fear or anticipatory anxiety awaiting the next bang. Previous studies indicated that some dogs react to loud noise by decreasing activity, others with an increase in activity^45^. Besides the possible difference in active vs passive coping styles^45^, the diverging results of previous studies regarding changes in activity when dogs were exposed to noise recordings could possibly be explained by the novelty of the environment: In a novel environment (such as during open field tests^42,43^), exploration would be expected in the beginning of the test. Consequentially, startling stimuli could be expected to inhibit exploration, and thus activity. On the other hand, after animals have habituated to the surroundings or if they are familiar with the environment such as in the present study, they might be more likely to spend the time resting, and so an increase in activity, as in the current study, might be noted as a result of fear-inducing events.

Panting was significantly associated with the fireworks condition in the current study, with eight of the nine dogs who panted doing so only during the fireworks. We consider panting in this situation to be fear-related, as the increased locomotion observed at the group level during fireworks cannot account for the high amount of time panting in the eight dogs that panted: four of these dogs remained stationary throughout the video, and only two were moving for more than ten percent of the time. Similarly, in a previous study, panting and pacing constituted differing coping strategies employed by different dogs^22^.

While the primary function of panting lies in thermoregulation^55^, panting is commonly regarded as an indicator of short-term stress, fear or anxiety in dogs^37,39,56,57^. One possible explanation for the mechanism underlying this association might be the need to dissipate heat, since stress leads to an increase in core body temperature, as commonly reported in dogs^58^ and other mammalian species (reviewed in^59^). Panting has been described as a consequence of the rapid activation of the autonomic nervous system, along with piloerection and trembling^60^ and may be a response associated with physiological arousal due to perceived external stimuli in dogs^56^. As in the current study, only a subset of dogs have been observed to pant during stressful situations, one contributing factor being dog size. Pastore et al.^56^ found a higher incidence of panting in female and larger dogs during a high-arousal situation, the latter being consistent with panting as a thermoregulatory response due to stress-induced hyperthermia, given that smaller dogs will dissipate relatively more heat from their bodies than bigger dogs due to their larger surface/volume ratio (c.f.^61^).

However, some contradictory results regarding panting in dogs in relation to stressful situations have been found. Although panting is often increased in fear-inducing situations^22,39,51^, panting in young dogs < 6 months was reduced during exposure to fear-inducing stimuli relative to control trials^33^, and dogs that were separated from their owner showed an increased frequency of panting upon reunion with their owner^62^. Panting might thus be indicative of both positive and negative arousal (c.f. ^56^). These behavioural signs thus need to be interpreted in the context in which they are occurring and in conjunction with other indicators.

The frequency of vocalisations was higher during the fireworks than during the control night (after correction for multiple testing, this was only a trend), but as with panting, this effect was driven by just nine individuals – the majority of subjects did not vocalise at all during either condition. Like panting, vocalisations can occur in contexts unrelated to fear, such as during greeting, play initiation, submission, defence, threatening behaviour, contact seeking, pain, or loneliness (reviewed in^63^). Thus, while panting and vocalising in a putatively fear-inducing situations may signify fear (e.g.^43,45,46,52^), they can also be shown for various other reasons. Conversely, we cannot draw the conclusion that there is no fear in the absence of these signs. For example, Overall et al.^22^ reported that dogs of different breeds may respond differently to loud noises (e.g. German shepherds often reacted by pacing, while Border collies and Australian shepherds showed a high rate of hiding and panting). Also individuals within a single breed may adopt different coping strategies associated with either active or passive (and more subtle) coping behaviours^43,44^.

Taken together, both the sensitivity and the specificity of vocalisations and of panting as fear indicators are low; i.e. not all fearful dogs vocalise or pant, and not all dogs that vocalise or pant are fearful. It is important to take such individual behavioural differences into account when making an assessment of an individual’s level of fear. Therefore, since relevant measures may not be the same for all individuals, it is likely that the assessment of the animal’s putative emotional state can be improved by considering evidence from several different indicators as well as the context (c.f.^3^).

In line with the suggestion by Mills^52^ that an elevated blinking rate may be associated with fear in dogs, blinking tended to be higher during the fireworks night compared to the control night (not significant after correction for multiple testing). In view of large inter-individual baseline differences in this measure and the need to correct for multiple testing, our study may have been underpowered for detecting a significant effect – the effect size was moderate at Cohen’s d = 0.38. Blinking constitutes a component of the startle reflex^64^, which could potentially explain the higher rate of blinking during fireworks. However, it has also been considered as an indicator of stress and/or anxiety per se in humans (e.g.^64–68^). Moreover, in dogs, Bremhorst et al.^5^ found an increase in blinking frequency when dogs were frustrated. This might indicate that an elevated blinking rate may constitute a sign of stress in dogs, rather than being specific to the emotions of either fear or frustration. Perhaps one difficulty of using this indicator in an applied setting lies in the fact that blinking is also a normal mammalian behaviour necessary to prevent dry eyes^69^, and so it would be necessary to identify deviations from baseline rates in order to draw conclusions about possible underlying affective states. Notably, the inter-individual variation in blinking rate also during the control condition was very high in our study (mean: 3.83/min, SD 4.07).

Dogs tended to hide more during the fireworks night than during the control night (non-significant after correction for multiple testing). Other studies similarly reported hiding in dogs that were exposed to loud noises, but as in our study there appeared to be much variation in whether dogs adopted this coping strategy^22,39,40^. A convenience sample was used in the current study. While the dogs in the current study were rated as mostly fearful by their owners (see Methods), in a study on dogs that were diagnosed with noise phobia, proportions of dogs showing hiding (85%) and panting (67%, as reported by the dogs’ owners) were higher than in the current study. Since our analysis was based on just three minutes of video material, it is possible that these behaviours, if they occurred, were not captured in the videos analysed, or they might be characteristic of more severe fear responses, as also indicated by de Souza et al.^40^. In contrast, more subtle indicators such as ears back might be more ubiquitously shown in fearful situations.

Our data show that even very strong fear-inducing non-social stimuli, fireworks, are not associated with an elevation in the rate of the so-called ‘stress signals’ lip licking or yawning in dogs. Similarly, de Souza et al.^40^ did not find an increase in lip licking and yawning in dogs exposed to recordings of a thunderstorm. Also, less severe fear-inducing non-social stimuli were unrelated to rates of lip licking^33,34^ or yawning^33^. In Stellato et al.^34^, subtle behaviours, including lip licking and yawning, were not affected by a non-social stimulus, but moderate correlations were found with the level of avoidance shown by dogs upon appearance of a masked stranger and when approaching the stranger. However, evidence was not conclusive, as there was much individual variation in these behavioural signs, the frequency per individual was low, and not all dogs rated as fearful based on gross behavioural measures showed these subtle behaviours at all^34^. Our results affirm the conclusion by Stellato et al.^34^ that these expressions do not represent good measures of fear in dogs, at least not with regard to non-social stimuli. Alternatively, they might play a role in social communication. Lip licking and yawning have been considered to function as appeasement signals both inter^54^- and intraspecifically^70^, and lip licking appears to be shown in situations of mild social threat (but less so during severe threats)^54^, as well as in greeting situations^54,71,72^.

### Conclusions

To our knowledge, this is the first study objectively measuring behavioural expression of pet dogs in their home settings during exposure to real-life fireworks, an approach which has advantages and drawbacks. Thus, due to using a citizen science approach, it was not possible to achieve complete standardisation and control, such as regarding firework intensity, owner behaviour and possible variation besides fireworks between the firework and the control situation. We made the decision not to restrict owners’ behaviour for ethical reasons to avoid imposing additional fear on dogs that might normally gain comfort from their owners, but also in view of having a real-life scenario, the latter being an advantage of the current study. Despite the fact that the study was not perfectly controlled, we are confident that the fireworks were the most salient arousing stimulus during the firework videos, as we discerned no presence of guests or unusual behaviours by the participating owners. Importantly, the observed behavioural differences shown during fireworks as compared to the control night are consistent with behavioural signs reported previously for noise fears in dogs, providing external validation to scales to assess noise fears in dogs where these behavioural signs are included (e.g.^73^) and strengthening the assumption that the measured behaviours are indeed fear-related.

Nonetheless it has to be acknowledged that identifying behavioural indicators of emotions in dog is challenging, as on the one hand, domestic dogs have a very rich behavioural repertoire, on the other hand, similar expressions may be shown in different contexts, and different individuals may react differently to fear-inducing situations, as indicated in this and previous studies. Based on the component process model of emotions, inferences about the probable emotion experienced by nonhuman animals can be made based on the combined analysis of the appraisal component (presence of stimuli that may be emotionally relevant for the animal), the arousal component (e.g. heart rate), action tendency, and finally behavioural expression^3^. Thus, in future studies, the identification of behavioural emotion indicators could be strengthened further by including an objective measurement of the arousal component, such as via heart rate or cortisol measurements. While it may often not be feasible in clinical practice to obtain physiological measurements to gauge dogs’ arousal associated with fear reactions in their home environment, collecting as much information as possible regarding the appraisal component (emotional relevance of the situation; in this case firework noises, which are perceived as threatening by many dogs), action tendency (e.g. escape attempts), and a variety of different expressive behaviours, may constitute the best method to infer the likely underlying emotion experienced by nonhuman animals.

## Methods

A citizen science study was performed. Volunteer dog owners were recruited via social media, a cynological association and dog sports groups. The owners were asked to film their dog for five minutes (1) during fireworks on New Year’s Eve (firework video) and (2) again several days later at a similar time in the evening (control video) of what they considered their dog’s normal behaviour. The time gap between the two video recordings could not be entirely standardised due to the citizen science approach, but the majority of participants sent in the second video within one week of New Year’s eve, and two participants within one month. The participating owners were sent an instruction sheet, detailing that both videos should be taken indoors and under good light condition, with the camera approximately one meter apart from the dog to avoid causing any stress by filming at a too close proximity. Owners were instructed to film one dog at a time and that they should aim to have the full body in view.

Prior to participation, owners were asked to fill in a questionnaire about their dogs’ behaviour during fireworks and to rate the ‘Welfare Impaired score’, deduced from the following statement “Please rate your level of agreement with the following statement: The overall welfare of my dog is strongly compromised by fireworks”, which could be answered on a five-point Likert scale from “disagree strongly” (1) to “agree strongly” (5), c.f.^31,32^. We excluded dogs from the study if the owners reported no welfare impairment at all during fireworks (Impaired Welfare Score of 1) to ensure the highest likelihood that the included dogs analysed were indeed fearful, considering that a previous study demonstrated high correlations between caregivers’ perceptions of their dogs’ sound sensitivity and behavioural indices derived from video coding of behaviour during exposure to a noise recording^40^.

For ethical reasons, no dog was exposed to additional stress. Thus, the owners were advised to carry out all measures they would normally take in an effort to alleviate their dogs’ stress, and to behave as they normally would during fireworks. This could include petting or feeding their dogs as well as using pheromones, homeopathic or herbal remedies, supplements, Bach flowers or aromatherapy. Since a previous study indicated that solely psychoactive medication, but none of the alternative products, led to a considerable improvement in firework fears^31^, only dogs receiving anxiolytic medication and dogs with known loss of hearing were excluded from the study.

The subjects were of various ages, breeds, sizes and both sexes, including 17 mixed breed dogs and 19 pure breed dogs of 15 different breeds (Supplementary Table S2). Eighteen dogs were female (5 intact, 13 neutered) and the same number were male (5 intact, 11 neutered, two chemically castrated), with an age range of six months to twelve years. The median ‘Welfare Impaired score’ of the 36 dogs was 4 (IQR: 2.75–5).

Two dogs were eating/licking a kong for the majority of time during the fireworks video (one of which did the same during the control video). Four dogs showed eating for a few seconds only. Fourteen dogs were petted during part of the time during the firework videos (six of which for more than one minute), while during the control videos five dogs were petted (one for more than one minute). Although we did not specifically ask about the identity of the people present during the videos, no more than two people (including the filmer) could be discerned in any of the submitted videos.

### Ethical consideration

The study was assessed and approved by the cantonal authority for animal experimentation, the Veterinary Office of the Canton of Bern (Switzerland) (Licence number BE28/17) and complies with the «Guidelines for the Treatment of Animals in Behavioral Research and Teaching» of the Association for the Study of Animal Behavior (ASAB). All participating owners gave their informed consent to the use of their videos for scientific analysis.

### Coding

As not all the submitted videos were of the requested duration of five minutes, but most videos were at least 3 min long, it was decided to code the first three minutes of each video. Some videos (in particular, the control videos where dogs were sleeping) were shorter. Thus, the mean duration ± SEM coded of the firework videos was 172.38 ± 4.19 s, and the mean duration ± SEM of the control videos was 147.86 ± 8.03 s. Videos were coded using Solomon Coder (András Péter; www.solomoncoder.com). The coder was blinded as to whether the videos were recorded during fireworks or control nights; however, in some of the videos it could be guessed which situation was filmed based on the context (e.g. presence of champagne glasses).

The frequency of events including blinking, snout licking, lip smacking, yawning, whining, howling and barking and the durations of sleeping, panting and different “invisible” categories (whole dog invisible, body invisible, head invisible, eyes invisible, and mouth invisible) were coded (see Table 1 for definitions). The “sleeping” category and the different “invisible” categories allowed us to calculate the frequencies of behaviours per minute while the behaviour was observable. Thus, the final frequency of blinking per minute was calculated as number of blinks recorded divided by time the eyes were visible (total time minus durations of sleeping, whole dog invisible, head invisible, and eyes invisible) and multiplied by 60. To calculate the frequency of oral behaviours per minute, the coded frequency was divided by total time minus the sum of time sleeping, whole dog invisible, head invisible and mouth invisible and multiplied by 60. Gross behaviours, moving and hiding, were coded as point samples every five seconds and converted to proportions of time. Ear position, body posture and tail position were scored every five seconds using a five-point scale (Table 1), and the mean score over all sampling points was calculated.

#### Reliability

A second coder coded the first 60 s of randomly selected videos of 20 dogs (10 firework videos, 10 control videos). Because some rarer behaviours were not shown at all in the randomly selected videos (panting, hiding), reliability coding for these variables was subsequently repeated based on a non-random selection of 13 videos, which included all videos in which either panting or hiding occurred at all according to the first coder’s codings. Cronbach’s alpha was calculated to assess reliability of the frequency of events per minute, the proportion of time spent in different behavioural states and the means of the posture, ear and tail scores (as mean values over all scores per dog were used as dependent variables, the scores were on a continuous scale and so Cronbach’s alpha was appropriate also for the scores). Reliability was good at α = 0.70 or higher for all variables included in the analysis (Supplementary Table S3).

### Analysis

Data were analysed using Statistica 6.1. (Statsoft Inc. 1984–2004) and IBM SPSS Statistics Version 23 (IBM Corporation and its licensors 1989, 2015).

#### Selection of variables

The variables ‘body posture’ and ‘tail position’ could not be included in the analysis, as dogs were mostly in a lying position during the control condition, so data from both conditions were available for only five (‘body posture) and three (‘tail position’) out of the 36 subjects, respectively. As vocalisations were rare (barking: N = 6; whining or howling: N = 5), frequencies of barking, whining and howling were added up to yield a single ‘vocalisation’ variable (Table 1). The frequency of snout licks and of lip smacks was very highly correlated, both during the firework condition (Spearman rank correlation test, N = 36, R_S_ = 0.75, p < 0.0001) and the control condition (N = 35, N = 36, Rs = 0.75, p < 0.0001); therefore, we created a composite variable labelled ‘lip licks’ (defined as the sum of ‘snout licks’ and ‘lip smacks’) to be used in further analyses. One dog was sleeping throughout the control condition, and therefore no frequencies of oral behaviours were coded for this dog during this condition. Thus, the following variables were compared between the fireworks and the control condition: ear position, frequency of blinking/min, lip licks/min, yawns/min, vocalisations/min, proportion of time panting, proportion of time moving and proportion of time hiding.

As most variables did not fulfil the requirements of parametric analysis, Wilcoxon signed rank tests were used to compare behaviours and scores between firework and control videos. As a measure of effect size, Cohen’s d^74^ was calculated as d = Z/√N (c.f.^75^).

#### Correction for multiple testing

To be able to gauge the significance of the results in view of the multiple statistical comparisons, we include the corrected alpha level according to sequential Bonferroni correction in Supplementary Table S1.

## Supplementary information

Supplementary file1

## Data Availability

The dataset generated and analysed during the current study is available from the corresponding author on reasonable request.
