# Supplementary material for: Fear expressions of dogs during New Year fireworks: a video analysis
Source: Sci Rep. 2020 Sep 29;10:16035. doi: 10.1038/s41598-020-72841-7 (PMC7525486; doi:10.1038/s41598-020-72841-7)
Supplement: Supplementary file 1 — Supplementary file1 [file 41598_2020_72841_MOESM1_ESM.docx]

**Video analysis of dogs' fear responses during New Year fireworks**

**Sarah Gähwiler^1^, Annika Bremhorst^1,2^, Katinka Tóth^3^, Stefanie Riemer^1,^***

^1^ Companion Animal Behaviour Group, Division of Animal Welfare, Vetsuisse Faculty, University of Bern, 3012 Bern, Switzerland

^2^ Animal Behaviour Cognition and Welfare Group, School of Life Sciences, University of Lincoln, Lincoln, LN6 7DL, United Kingdom

^3^ Research Centre for Natural Sciences, Institute of Cognitive Neuroscience and Psychology, Budapest, Hungary

* [riemer.stefanie@gmail.com](mailto:riemer.stefanie@gmail.com)

**Supplementary information**

**Supplementary Table 1.** Demographic information about the subjects and owner-assessed welfare impaired score

| **ID** | **Breed** | **Age** | **Sex** | **Neuter status** | **Welfare impaired score** |
| --- | --- | --- | --- | --- | --- |
| 1 | Mixed breed | 4.11 | Female | Neutered | 4 |
| 2 | Mixed breed | 8.99 | Male | Neutered | 2 |
| 3 | German shepherd | unknown | Male | Intact | 2 |
| 4 | Flat coated retriever | 10.94 | Male | Intact | 2 |
| 5 | French bulldog | 0.43 | Female | Intact | 2 |
| 6 | Swiss mountain dog | 9.75 | Male | Neutered | 5 |
| 7 | Coton de Tuléar | 8.88 | Female | Neutered | 3 |
| 8 | Border collie | 6.32 | Male | intact | 4 |
| 9 | Mixed breed | 3.27 | Male | Neutered | 5 |
| 10 | Mixed breed | 9.42 | Male | Neutered | 3 |
| 11 | Mixed breed | 3.92 | Female | Neutered | 5 |
| 12 | Yorkshire terrier | 1.11 | Male | Neutered | 4 |
| 13 | Mixed breed | 6.42 | Male | Neutered | 5 |
| 14 | Shiba inu | 3.46 | Female | Intact | 5 |
| 15 | Mixed breed | 6.69 | Male | Neutered | 5 |
| 16 | Golden Retriever | 0.74 | Female | Intact | 2 |
| 17 | Chinese Crested Powderpuff | 3.44 | Female | Neutered | 3 |
| 18 | Mixed breed | 1.9 | Female | Neutered | 3 |
| 19 | Mixed breed | 4.34 | Male | Neutered | 3 |
| 20 | Labrador Retriever | 6.63 | Female | Intact | 4 |
| 21 | Mixed breed | 2.67 | Female | Neutered | 5 |
| 22 | Mixed breed | 8.59 | Female | Neutered | 4 |
| 23 | Mixed breed | 9.33 | Male | Neutered | 4 |
| 24 | Mixed breed | 6.67 | Male | Neutered | 4 |
| 25 | German Pinscher | 8.45 | Female | Neutered | 2 |
| 26 | Mixed breed | 10.64 | Male | Intact | 4 |
| 27 | Mixed breed | 3.58 | Female | Neutered | 4 |
| 28 | French bulldog | 0.43 | Female | Intact | 3 |
| 29 | Golden Retriever | 3.61 | Male | Chemical castration | 2 |
| 30 | Mixed breed | unknown | Female | Neutered | 2 |
| 31 | Mixed breed | 12.6 | Female | Neutered | 2 |
| 32 | Magyar vizsla | 9 | Female | Neutered | 5 |
| 33 | Border collie | 4.91 | Male | Intact | 5 |
| 34 | Australian Shepherd | 3.67 | Male | Chemical castration | 4 |
| 35 | German shepherd | 7.45 | Male | Neutered | 5 |
| 36 | Giant schnauzer | 9.24 | Female | Neutered | 5 |

**Supplementary Table 2.** Median and interquartile range (IQR) for behaviours shown by dogs during firework and control videos and results of a Wilcoxon signed ranks test comparing those during the two conditions. The corrected alpha level according to sequential Bonferroni correction is indicated next to the original p value. Cohen’s d is included as a measure of effect size.

| **Variable** | **Median (IQR) – Fireworks** | **Median (IQR) – Control** | **Z** | **p** | **Seq. Bonferroni-corrected alpha level** | **Cohen’s d** |
| --- | --- | --- | --- | --- | --- | --- |
| Ear position | 2.27  (1.80-2.86) | 3.00  (1.72-5) | 4.05 | 0.00005 | 0.0062 | 0.68 |
| % time moving | 4.17  (0.00-13.39) | 0.00  (0.00-0.00) | 3.24 | 0.001 | 0.0071 | 0.54 |
| % time panting | 0.00  (0.00-10.60) | 0.00  (0.00-0.00) | 2.67 | 0.008 | 0.0083 | 0.45 |
| Vocalisation/ min | 0.00  (0.00-0.00) | 0.00  (0.00-0.00) | 2.37 | 0.018 | 0.01 | 0.40 |
| Blink/ min | 6.58  (2.51-9.57) | 3.11  (0.68-6.00) | 2.23 | 0.026 | 0.0125 | 0.37 |
| % time hiding | 0.00  (0.00-0.00) | 0.00  (0.00-0.00) | 2.2 | 0.028 | 0.0167 | 0.37 |
| Yawn/ min | 0.00  (0.00-0.00) | 0.00  (0.00-0.35) | 1.22 | 0.221 | 0.025 | 0.21 |
| Lip lick/ min | 0.91  (0-4.00) | 0.00  (0.00-2.88) | 0.74 | 0.46 | 0.05 | 0.13 |

**Supplementary Table 3.** Inter-rater reliability (Cronbach’s α) for the analysed behaviours.

| **Variable** | **Cronbach’s α** |
| --- | --- |
| Ear position | 0.70 |
| Blink/ min | 0.92 |
| Lip lick/ min | 0.92 |
| Yawn/ min | 0.99 |
| Vocalisation/ min | 1.00 |
| % time panting | 0.98 |
| % time moving | 0.82 |
| % time hiding | 0.99 |
